# Toxic Effects of Prodigiosin Secreted by *Hahella* sp. KA22 on Harmful Alga *Phaeocystis globosa*

**DOI:** 10.3389/fmicb.2017.00999

**Published:** 2017-06-06

**Authors:** Huajun Zhang, Hui Wang, Wei Zheng, Zhiyuan Yao, Yun Peng, Su Zhang, Zhong Hu, Zhen Tao, Tianling Zheng

**Affiliations:** ^1^School of Marine Sciences, Ningbo UniversityNingbo, China; ^2^Biology Department, College of Life Science, Shantou UniversityShantou, China; ^3^State Key Laboratory of Marine Environmental Science and Key Laboratory of the Ministry of Education for Coastal and Wetland Ecosystems, School of Life Sciences, Xiamen UniversityXiamen, China

**Keywords:** prodigiosin, *Phaeocystis globosa*, oxidative stress, photosynthesis inhibition, cell death

## Abstract

Application of algicidal compounds secreted by bacteria is a promising and environmentally friendly strategy to control harmful algal blooms (HABs). Years ago prodigiosin was described as an efficient algicidal compound, but the details about the effect of prodigiosin on algal cells are still elusive. Prodigiosin shows high algicidal activity on *Phaeocystis globosa*, making it a potential algicide in HAB control. When *P. globosa* were treated with prodigiosin at 5 μg/mL, algae cells showed cytoplasmic hypervacuolization, chloroplast and nucleus rupture, flagella missing, and cell fracture, when observed by scanning electron microscope and transmission electron microscopy. Prodigiosin induced a reactive oxygen species (ROS) burst in *P. globosa* at 2 h, which could result in severe oxidative damage to algal cells. Chlorophyll *a* (Chl *a*) fluorescence decreased significantly after prodigiosin treatment; about 45.3 and 90.0% of algal cells lost Chl *a* fluorescence at 24 and 48 h. The *F*_v_/*F*_m_ value, reflecting the status of the photosystem II electron flow also decreased after prodigiosin treatment. Quantitative polymerase chain reaction (PCR) analysis *psb*A and *rbc*S expression indicated that photosynthesis process was remarkably inhibited by prodigiosin. The results indicated that the inhibition of photosynthesis may produce excessive ROS causing cell necrosis. This study is the first report about algal lysis mechanism of prodigiosin on harmful algae. Our results could increase our knowledge on the interaction between algicidal compounds and harmful algae, which could lead to further studies in the microcosm.

## Introduction

Harmful algal blooms (HABs), as the most severe consequence of eutrophication, occur worldwide causing irreversible damage to fisheries, tourism, public health, and the ecosystem (Heisler et al., 2008). *Phaeocystis* (Prymnesiophyceae), an important widespread marine haptophyte, could lead to blooms with agminated colonies in marine ecosystem (Rousseau et al., 2007). HAB, caused by *Phaeocystis globosa*, are frequent blooms in coastal waters (Sheik et al., 2014), producing excessive foam when elimination, which would be a problem for socio-economic activities (Peperzak and Gäbler-Schwarz, 2012).

Continual HAB outbreaks in coastal waters had received the focus of scientists, promoting to develop innovative technologies and strategies to management it (Anderson et al., 2012). There are several strategies to control HABs, including the use of physical methods (i.e., clay) and chemical algicides (Li and Pan, 2013; Guo et al., 2015). In recent years, biological methods to control HAB have got special attention on account of their species specificity, effectiveness, and eco-friendly properties (Roth et al., 2008; Son et al., 2015). Interestingly, some bacteria are able to secrete metabolic compounds specifically killing harmful phytoplankton, suggesting that the algicide from bacteria might function as a harmful algae biological control agent in seawaters (Wang et al., 2010a). In the past, we had identified an effective algicidal compound, prodigiosin, from *Hahella* sp. KA22 that shows high algicidal activity against *P. globosa* (Zhang et al., 2016). Prodiginines (6-methoxyprodigiosenes) comprise of various pigmented tripyrrole antibiotics, with potential medical use as immunosuppressants and antitumor agents (Sang et al., 1998; Pérez-Tomás et al., 2003), that can also kill red tide *Cochlodinium polykrikoides* dinoflagellates (Jeong et al., 2005). It has been proved that prodigiosin possesses algicidal activity particularly on several specific dinoflagellates, including *C. polykrikoides*, *Gyrodinium impudicum*, and *Heterosigma akashiwo*, and also raphidophyte *Chattonella* sp. (Kim et al., 2008). Our previous study demonstrated that prodigiosin could lyse *P. globosa* cells at the concentration of 1 μg/mL (Zhang et al., 2016). Moreover, the prodigiosin was thermo-stable and light-degradable indicating that prodigiosin may be a potential algicide for controlling HAB in natural environments. However, the activity of prodigiosin has not been thoroughly examined and the algicidal mechanism on *P. globosa* remains elusive.

Current studies indicate that the algicidal mechanisms against HAB include mainly four pathways: cell structure destruction, photosynthesis inhibition, respiration impairment, and alteration of enzymatic activities (Choudhary et al., 2007; Affenzeller et al., 2009; Zhang et al., 2010). During the algicidal process, the algicidal compounds could induce various damage (i.e., oxidative) to algal cells, morphological alterations, and degradation of DNA or proteins that can result in cell death. Aquatic organisms, including algae, possess antioxidant defenses system to remove excessive reactive oxygen species (ROS) and thus prevent oxidative damage triggered by malondialdehyde (MDA). Catalase (CAT), superoxide dismutase (SOD), glutathione peroxide (GPx), glutathione reductase (GR), and micromolecular compounds including carotenoids and glutathione, constitute antioxidant defenses system (Kirilovsky, 2015; Sanchez et al., 2015). Therefore, study on the physiological response of algae to algicidal compounds may be helpful to understand their algicidal mechanisms.

Our previous study was mainly on the isolation and characterization of prodigiosin from *Hahella* sp. KA22 (deposited in China Center for Type Culture Collection; CCTCC M2014297). We demonstrated that prodigiosin could efficiently kill *P. globosa* (Zhang et al., 2016). The present study provides the first evidence about the algicidal mechanism of this pigment on *P. globosa.* With the purpose to explore the algicidal mechanism of prodigiosin, the present study were designed to (1) investigate the alteration of algal cell structure including membrane, nucleus and ultrastructure; (2) study the response of antioxidant system in algal cells; and (3) explore photosynthesis damage of algal cells.

## Materials and Methods

### Algal Culture Preparation and Prodigiosin Treatment

*Phaeocystis globosa* culture was stored in the Algal Culture Collection (Jinan University), under the accession number PG03. Algae were incubated in sterile f/2 medium, and cultured at 20 ± 1°C under a 12 h light:12 h dark cycle with a 50 μmol photons m^-2^ s^-1^ light intensity (Zheng et al., 2012). Algal cells in the logarithmic growth phase (10^6^ cells/mL) were prepared for all the following tests.

Prodigiosin dissolved in dimethylsulfoxide (DMSO) was added into 20 mL algal cultures to the final concentrations of 3 and 5 μg/mL. Sampling was performed at 12, 24, 36, 48, and 72 h, by centrifugation at 5,000 × *g* for 5 min. DMSO serving as control was also added into algal cultures with equal volume as prodigiosin.

### The Assays of ROS Changes, Malondialdehyde Content, and Antioxidative Enzyme Activities

ROS accumulation was analyzed by the fluorescent probe, 2′,7′-dichlorofluorescin diacetate (DCFH-DA), referring to a previously reported method (Rastogi et al., 2010) with some changes: 0.5 mL DCFH-DA were added to the cells and the mixture at the final concentration of 10 μM, then the mixture was stored at 37°C with light avoidance for about 1 h. Cells were then washed three times using sterile f/2 medium following stored in it. A spectrofluorometer was used to monitor the fluorescence intensity of the DCFH-DA with an excitation wavelength set at 485 nm and an emission wavelength at 525 nm.

The antioxidant system was also analyzed by measuring SOD, CAT, GR, and GPx in *P. globosa* according to methods described previously (Zhang et al., 2013). Twenty milliliters of each culture including control and treatment group were collected to detect the alteration of the antioxidative enzymes. MDA, which is a by-product of lipid peroxidation, was analyzed at the same time.

### Analysis of the Algicidal Process by Electron Microscopy and Laser Scanning Confocal Microscope

To analyze the morphological features of the algicidal process, algal cells were collected and observed by scanning electron microscope (SEM). Algal cells (1 mL) were first fixed with glutaraldehyde (2.5%) and washed in phosphate buffered saline (PBS) solution (50 mM, pH 7.8); cells were then placed on cover slips and air-dried. After that, the slides were subjected to SEM (JSM6390, JEOL Co., Tokyo, Japan) analysis after dehydration. To analyze ultrastructural changes, algal cells were observed by a transmission electron microscope (TEM, JEM2100HC, JEOL Co., Tokyo, Japan) according to our previous study (Zhang et al., 2013).

Changes in nucleus structure were analyzed by confocal laser scanning microscope (CLSM, Zeiss LSM 780, Carl Zeiss, Jena, Germany). Algal cells were fixed with 1% paraformaldehyde, then washed and resuspended in PBS. Cells were then stained with 5 μg/mL 4′,6-diamidino-2-phenylindole (DAPI) for 15 min in the dark. After washing with PBS, algal cells were placed on slides for CLSM observation. DAPI fluorescence was read at 460 nm and represented with blue color on images. Chlorophyll fluorescence was monitored at 680 nm and represented with red color on images.

### Analysis of Cell Membrane Permeability

For cell membrane permeability analysis, algal cells in treatment group were collected after 3, 12, 24, 36, and 48 h, followed by PBS (50 mM, pH 7.4) washing. Cell membrane permeability was then analyzed by the fluorescent dye propidium iodide (PI; Invitrogen, United States) and detected by flow cytometry (FCM; Wang et al., 2010b). A total of 900 μL algal cell solution (about 10^6^–10^7^ cells/mL) stained with 100 μL of PI at final concentration of 10 μg/mL. The mixture were then stored for 15 min at room temperature in the dark, then analyzed by FCM according to our method reported previously (Zhang et al., 2015). About 10,000 cells were analyzed in each sample.

### Measurement of Fluorescence of Pigments and Chlorophyll

To analyze the contents of chlorophyll *a* (Chl *a*) and carotenoid after prodigiosin treatment, 5 mL algal cultures were prepared as described above. Algal pigments were then extracted with 90% ethanol at 4°C in the dark, overnight (Ritchie, 2006). After cells removed by centrifugation, the supernatants absorbance was tested at 665, 645, and 470 nm. Chl *a* and carotenoid content was calculated using the following formulas:

$$\text{Chlorophyll a }(\text{mg/L})=12.7*A_{665}-2.69*A_{645}$$

$$\text{Carotinoid }(\text{mg/L})=(1,000*A_{470}-2.05*C_{\text{Chlorophyll a}})/245$$

Chl *a* fluorescence was also detected by FCM as described above, but fluorescence intensity was collected using a 630-nm filter (FL4).

Pulse amplitude modulation (PAM) fluorescence indicating photosynthesis efficiency was measured by PAM-CONTROL Fluorometer (Walz, Effeltrich, Germany). The algal fluorescence was detected using an actinic light of 3,000 μmol photons m^-2^ s^-1^ after dark adaptation for 15 min. The maximum photochemical quantum yield of photosystem (*F*_v_/*F*_m_) which is the indicator of photosynthesis was obtained (Schreiber et al., 2007).

### The Photosynthesis-Related Genes Expression Assays

To analyze the effect of prodigiosin on the genes expression, total RNA was extracted from 50 mL algal cells treated for 12, 24, and 36 h using the RNAiso kit (TaKaRa Company). Two photosynthesis-related genes were analyzed using specific primers: 5′-AGTTGCTGGTTCTCTACTTTACG-3′ (forward) and 5′-TTCCCACTCA CGACCGATG-3′ (reverse) for the *psbA* gene; 5′-AAGTCTTACTGGGAAATGTG G G-3′ (forward) and 5′-AGCAGGACGCTGAACGATG-3′ (reverse) for the *rbcS* gene; 5′-TCCGATAACGAACGAGAC-3′ (forward) and 5′-TGACGCAAAC TTCCACTT-3′ (reverse) for the 18S rRNA gene. Quantitative real-time polymerase chain reaction (PCR) was performed under the following conditions: denaturation at 95°C for 3 min, then 40 cycles at 95°C for 10 s, and 55°C for 30 s. Meanwhile, 18S rRNA gene was employed as reference gene to normalize gene expression changes. Gene expression was quantified using the 2^-ΔΔCt^ method (Livak and Schmittgen, 2001).

### Statistical Analysis

All triplicate data analyzed with mean ± standard error and variance compared using one-way analysis of variance (ANOVA), followed by the least significant difference test (Origin 9.0 for Windows).

## Results

### Cell Morphology under Prodigiosin Treatment

**FIGURE 1:**
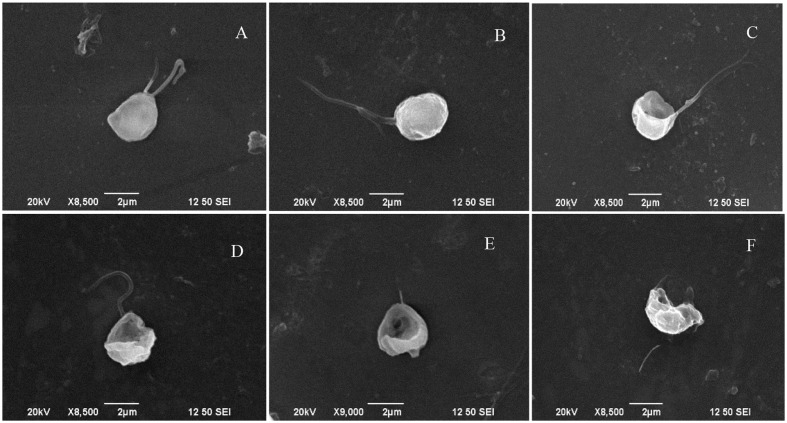
SEM micrographs showing the morphology of algal cells treated with prodigiosin. **(A,B)** Control group; **(C–F)** cells treated for 12, 24, 48, and 72 h, respectively. Scale bar = 2 μm.

It had been proved that 5 μg/mL prodigiosin produced 84% algicidal activity in 72 h (Zhang et al., 2016). In the present research, the algicidal process of prodigiosin on *P. globosa* was further analyzed by SEM (**Figure 1**). Normal algal cells exhibited one or two flagella (**Figures 1A,B**) indicating ability for high motility. When algae were treated with prodigiosin for 12 and 24 h, many cells had ruptured and had partially lost cell structure (**Figures 1C,D**). As exposure time increased, algal cells lysed and the cell structure was destroyed with cellular components released from cells, finally resulting in cell disintegration (**Figures 1E,F**). Flagella lost from the cells as exposure time increased, indicating that prodigiosin could affect cell motility.

### Nucleus Damage from Prodigiosin Treatment

**FIGURE 2:**
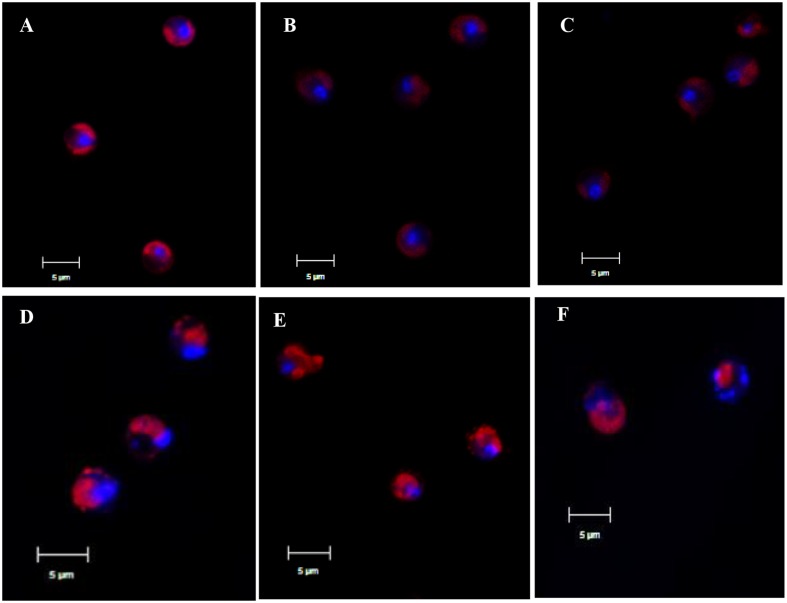
Representative CLSM micrographs of control algal cells **(A)** or cells treated with prodigiosin (5 μg/mL) for 12, 24, 36, 48, and 72 h **(B–F)**. Chlorophyll autofluorescence is shown in red, and the nucleus stained with 4′,6-diamidino-2-phenylindole (DAPI) is shown in blue. Scale bar = 5 μm.

CLSM revealed more details on the morphology of the nucleus after prodigiosin treatment (**Figure 2**). In control cells, chromatin is concentrated in the center of cells, as indicated by strong and even red autofluorescence and also DAPI blue emission (**Figure 2A**). However, chromatin displacement was observed after 24 h of prodigiosin treatment (**Figure 2C**), as algal cells became irregular. After 36 h of treatment, diffusion of the degraded DNA was observed (**Figure 2D**). As prodigiosin treatment time increased, the presence of DAPI fluorescence around the cells evidenced further DNA fragmentation and chromatin displacement (**Figures 2E,F**). Autofluorescence of chlorophyll also evidenced serious cell deformation (**Figures 2C–F**). Release of cellular inclusions and cell cleavage were similar to those observed by SEM analysis.

### Subcellular Structure Changes after Prodigiosin Treatment

**FIGURE 3:**
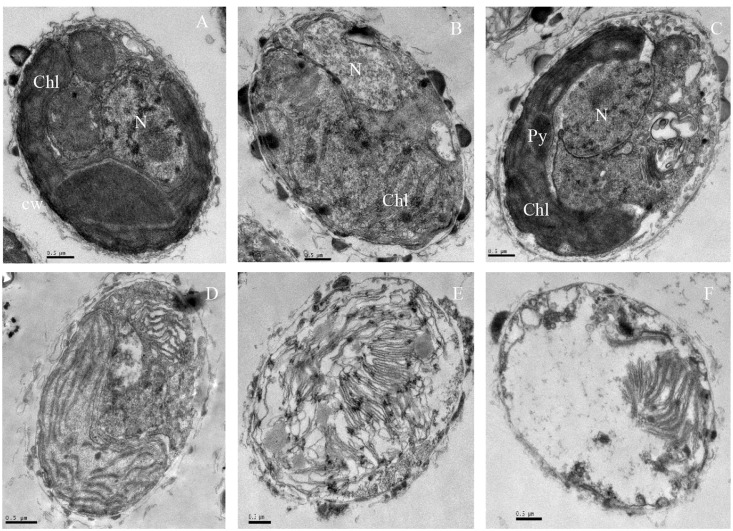
TEM micrographs showing the ultrastructure of algal cells treated with 5 μg/mL prodigiosin. **(A)** Control cells; **(B–F)** 12, 24, 36, 48, and 72 h of treatment; N, nucleus; Chl, chloroplast; Py, pyrenoid; Scale bars = 0.5 μm.

The ultrastructure of algal cells was compared between control cells and cells treated with 5 μg/mL prodigiosin (**Figure 3**). In control cells (**Figure 3A**), we could observe a normal nucleus, chloroplasts, mitochondria, and other organelles. After prodigiosin treatment, algal cells exhibited various structural differentiation, including morphological alterations and impairment. After 12 (**Figure 3B**) and 24 h (**Figure 3C**) of prodigiosin treatment, algal cells showed cytoplasmic hypervacuolization and were not as compact as control cells; however, the nuclear morphology was still normal at this time point. After 36 (**Figure 3D**) and 48 h (**Figure 3E**) of treatment, the vacuolization of the cytoplasmic intensified and many organelles broke up, including the chloroplasts and the nucleus. Algal cells treated with prodigiosin for 72 h (**Figure 3F**) showed extreme plasmolysis and vacuolization. Moreover, cellular inclusions released from cells, leaving a relatively intact cell wall, indicating that cells had completely lost vital activities.

### Integrity of Cell Membrane

**FIGURE 4:**
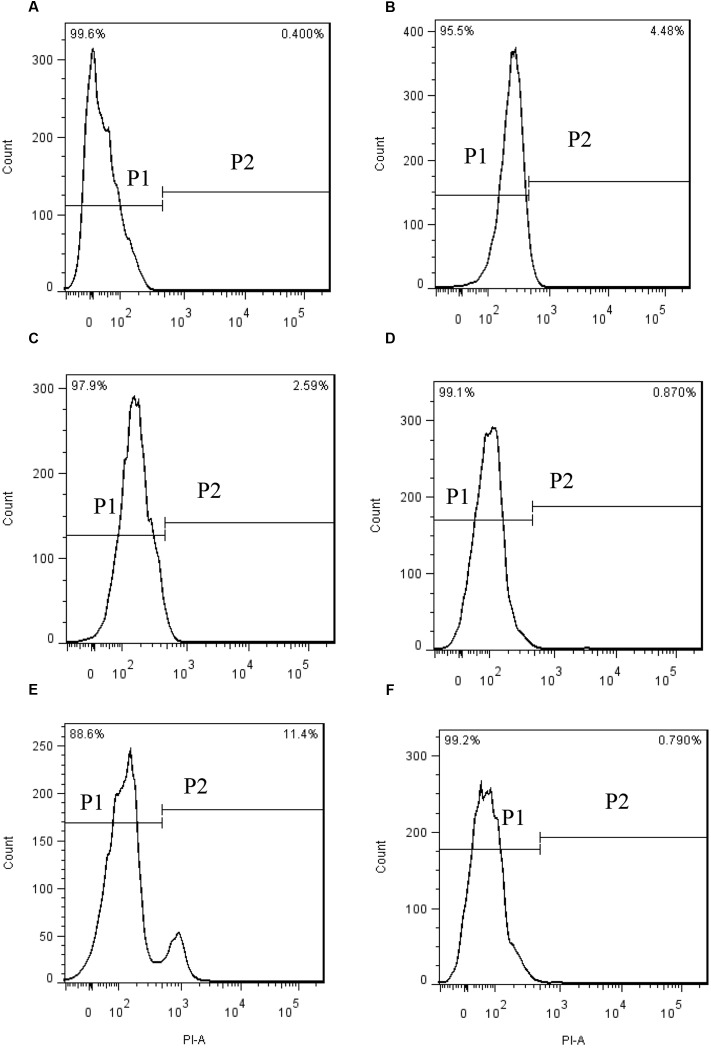
PI fluorescence density histogram indicating the integrity of algal cell membranes after different times of treatment with prodigiosin. **(A)** Control; **(B–F)** cells treated for 3, 12, 24, 36, and 48 h, respectively. Quadrant P1: cells without PI fluorescence. Quadrant P2: abnormal cells with PI fluorescence.

PI enters into living cells through permeabilized membranes, then intercalates into DNA or RNA emitting red fluorescence (Davey and Hexley, 2011). When algal cells were stained with PI after prodigiosin treatment, the fluorescence in the nucleus did not change significantly (**Figure 4**). The proportion of normal cells were 99.6% in the cytogram of untreated algal cells (**Figure 4A**, P1), while cells in P2 were permeabilized cells. Our data showed that most algal cells lysed in 48 h, but the membrane of remaining living cells was not affected by prodigiosin.

### Responses of Enzyme Activities to Prodigiosin Treatment

**FIGURE 5:**
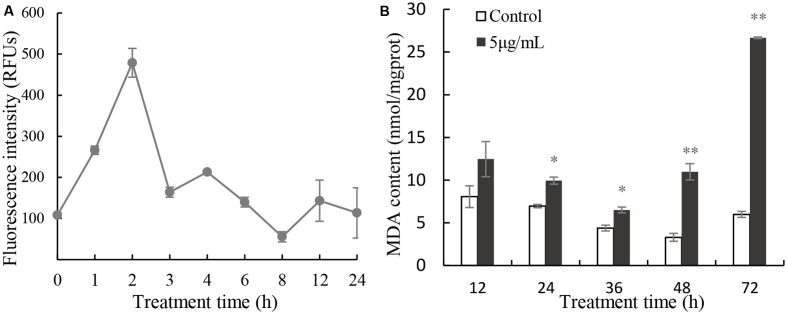
Intracellular ROS **(A)** production and MDA content **(B)** in cells treated with 5 μg/mL prodigiosin. Data are mean of three independent experiments ± SE, with the statistic of ^∗∗^*P* < 0.01 and ^∗^*P* < 0.05.

Excessive ROS can cause damage to molecules and cell structure. ROS increased rapidly after 2 h in cells treated with 5 μg/mL prodigiosin (**Figure 5A**) that were about fivefold higher than those of untreated cells. After that, ROS levels returned to a relative normal levels. To determine the cellular damage caused by ROS, we analyzed the MDA content which is a natural biomarker of the lipid peroxidation process (Sathasivam et al., 2016). MDA content in algal cells increased slightly after 36 h of exposure to prodigiosin, relative to the control (**Figure 5B**). After that, it increased remarkably (*P* < 0.01) after 48 h of treatment (3.32-fold; *P* < 0.01), reaching maximum value at 72 h of treatment (4.45-fold; *P* < 0.01).

**FIGURE 6:**
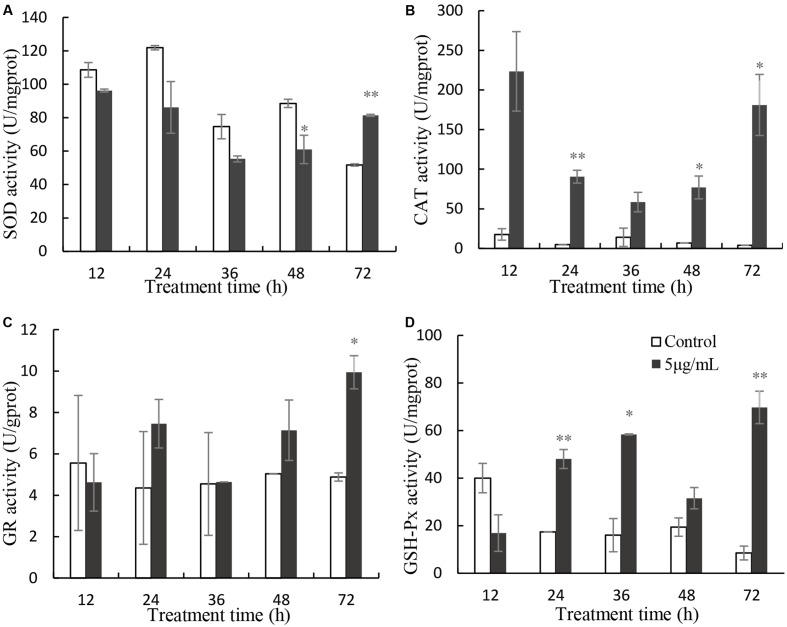
Effects of prodigiosin treatment (5 μg/mL) on the activity of SOD **(A)**, CAT **(B)**, GR **(C)**, and GPx **(D)**. Data are mean of three independent experiments ± SE, with the statistic of ^∗∗^*P* < 0.01 and ^∗^*P* < 0.05.

SOD activity decreased slightly, relative to the control after 12, 24, 36, and 48 h exposure, but it increased significantly after 72 h treatment (**Figure 6A**). The values of SOD were 0.89, 0.71, 0.74, 0.69 (*P* < 0.05) and 1.57 times (*p* < 0.01) those of the control at 12, 24, 36, 48, and 72 h treatment. CAT activity increased significantly, relative to the untreated cells (**Figure 6B**), after 12 h (12.64-fold), 24 h (18.52-fold; *P* < 0.01), 36 h (4.15-fold), 48 h (11.38-fold; *P* < 0.05), and 72 h (45.15-fold; *P* < 0.05) of exposure to 5 μg/mL prodigiosin. GR activity did not change markedly in the first 48 h of prodigiosin treatment, but increased after 72 h (2.04-fold; *P* < 0.05) treatment (**Figure 6C**). The effect of prodigiosin on GPx activity was similar to its effect on CAT activity (**Figure 6D**). After 24 h, and 36 h of treatment, GPx activity obviously increased (*P* < 0.01). Although GPx activity decreased at 48 h of treatment, it increased markedly (*P* < 0.01) after 72 h reaching its maximum.

### Algal Photosystem Responses

**FIGURE 7:**
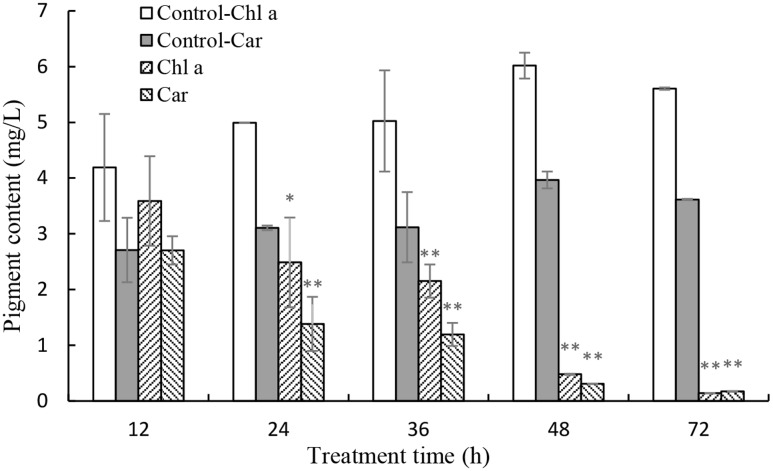
Effects of prodigiosin treatment (5 μg/mL) on the contents of Chl *a* and carotenoid. Data are mean of three independent experiments ± SE, with the statistic of ^∗∗^*P* < 0.01 and ^∗^*P* < 0.05.

**FIGURE 8:**
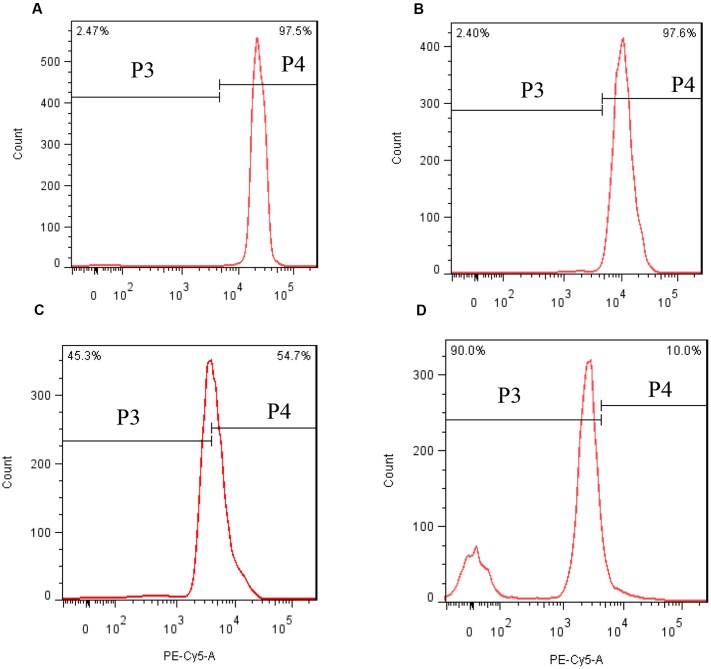
Chl *a* fluorescence in response to prodigiosin after different times of treatment. **(A)** Control; **(B–D)** 12, 24, and 48 h. Quadrant P4: Chl a fluorescence of normal cells. Quadrant P3: Chl a fluorescence of abnormal cells.

In order to investigate the stress of prodigiosin on the photosynthesis process, Chl *a* and carotenoid contents were determined. Chl *a* contents decreased, relative to untreated cells, after 12 h of exposure to prodigiosin (*P* < 0.05; **Figure 7**). As exposure time increased, Chl *a* contents decreased. Chl *a* contents in control were 12.6-fold at 48 h (*P* < 0.01), and 39.3-fold at 72 h (*P* < 0.01), relative to prodigiosin-treated cells. The carotenoids contents in algal cells changed in a way similar to Chl *a*. After 36, 48, and 72 h, carotenoids contents in control cells were about 2.60-, 12.75-, and 20.88-fold, respectively, relative to cells treated with prodigiosin. FCM analysis of Chl *a* fluorescence showed a remarkable change after prodigiosin treatment (**Figure 8**). About 97.5% of the untreated control cells had relative normal Chl *a* fluorescence (quadrant P4). Chl *a* fluorescence was not affected by prodigiosin in 97.6% of the cells after 12-h treatment (**Figure 8B**), but fluorescence decreased after 24 (**Figure 8C**) and 48 h (**Figure 8D**) of treatment. About 45.3% and 90.0% of algal cells lost Chl *a* fluorescence at 24 and 48 h of treatment, respectively.

**FIGURE 9:**
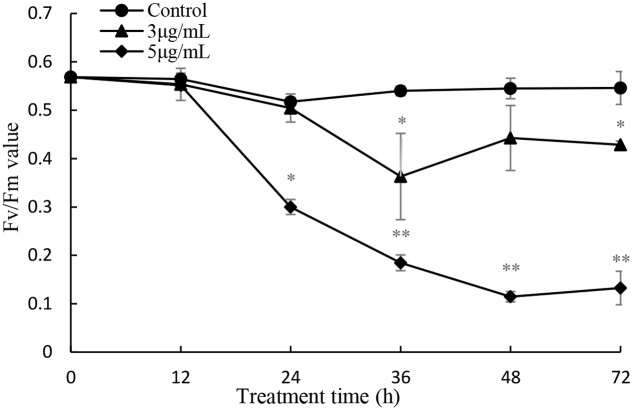
Influence of prodigiosin on photosynthetic efficiency (*F*_v_/*F*_m_) of *P. globosa.* Data are mean of three independent experiments ± SE, with the statistic of ^∗∗^*P* < 0.01 and ^∗^*P* < 0.05.

The maximum photochemical quantum yield (*F*_v_/*F*_m_) was employed to detect the photosynthetic status of the cells after prodigiosin treatment at 3 and 5 μg/mL (**Figure 9**). After 24 h of treatment, the *F*_v_/*F*_m_ value was remarkably (*P* < 0.05) lower than that of control cells when treated with 5 μg/mL prodigiosin. After 36 h of treatment, the *F*_v_/*F*_m_ value decreased significantly. *F*_v_/*F*_m_ values were lower in 5 μg/mL treatment group indicating that the inhibition in photosystem II (PSII) by prodigiosin was dose-depended.

**FIGURE 10:**
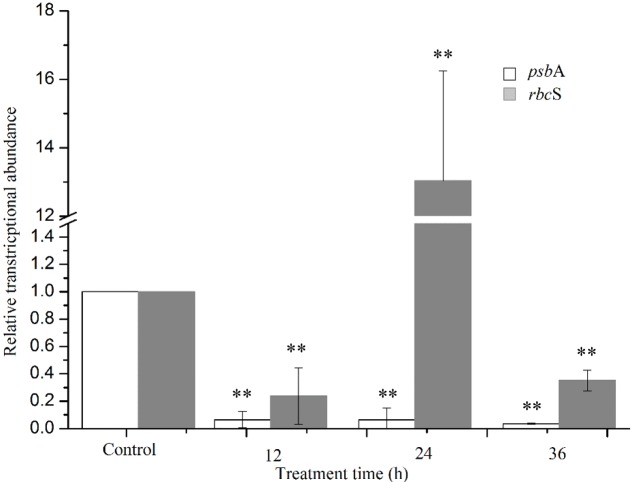
Relative normalized expression of *psb*A and *rbc*S genes in algal cells treated with 5 μg/mL prodigiosin. Data are mean of three independent experiments ± SE, with the statistic of ^∗∗^*P* < 0.01 and ^∗^*P* < 0.05.

Two target genes were investigated to explore the extent of damage to the photosystem (**Figure 10**). The *psbA* gene encodes the reaction center D1 protein of photosystem, which participates in the PSII photodamage repair process. Gene expression analysis was performed at 36 h of treatment, because RNA quality was not suitable for qPCR analysis after longer treatment times. The results showed that the *psbA* gene expression was significantly inhibited (*P* < 0.01) at all times of exposure, providing evidence for the inhibition of the photodamage repair process. The expression of *rbcS* gene reduced obviously (*P* < 0.01) after 12 h exposure, and then increased markedly (*P* < 0.01) after 24 h, indicating that algal cells could provide more energy to resist prodigiosin stress. As exposure time increased, prodigiosin continued to destroy cells and *rbcS* gene expression decreased again. These results indicate that the photodamage repair process was inhibited at 12 h, and that carbon fixation process was inhibited at 36 h. These results indicated that prodigiosin treatment induced photosynthesis inhibition at short time points, which may be finally related to algal cell death.

## Discussion

In our previous study, we found that strain KA22 could secrete prodigiosin exhibiting effective algicidal activity on *P. globosa* (Zhang et al., 2016). Prodigiosin possesses a huge advantage on the control of HAB because it could degrade naturally in environment avoiding persistence in marine waters (Zhang et al., 2016). In this work, we explored the mechanism of prodigiosin killing *P. globosa*, which will provide detail biological safety and ecological information for the usage of prodigiosin in environment in the future.

External stress can stimulate algal cells to generate excessive ROS, which could give rise to severe cellular damage or cell death. Our results showed that prodigiosin caused a ROS burst in *P. globosa* after a short time of treatment (**Figure 5**), which may ultimately lead to cell death. ROS could cause oxidative damage to algal cells, as evidenced by MDA contents (**Figure 5**), despite the activation of the antioxidant system (**Figure 6**). Increased ROS levels also induced DNA and nucleus damage, as evidenced by degraded chromatin, and a dispersed nucleus (**Figures 2**, **3**), which are features of necrotic cell death (Zong et al., 2004; Van Doorn et al., 2011). Moreover, ROS is well known to produce algal cells necrosis (Berghe et al., 2014).

It is known that exposure of unicellular alga to environmental stress induces disparate morphotypes of cell death (Jimenez et al., 2009). Few studies have focused on the ultrastructure of *P. globosa*, especially under external stress. We observed clear differences in ultrastructure between cells treated with prodigiosin and untreated cells, which indicated that prodigiosin produced necrosis (**Figure 3**). Cytoplasmic vacuolization, organelle decomposition, nuclear rupture, were typical characteristics of cell necrosis that were observed by TEM in this study. Our findings suggest that prodigiosin promoted some cells to undergo necrosis via ROS. To our knowledge, this is the first report for the necrosis of *P. globosa* cells induced by an algicidal pigment.

Chl *a* fluorescence analyzed by FCM was employed to detect response of photosynthesis to external stress. In fact, this method is frequently applied in toxicological and eco-physiological studies to detect the consequences of environmental alteration and pollutants on algae and higher plant. Prodigiosin showed obviously inhibitory effect on Chl *a* fluorescence after long exposure time, which may be the result of the inhibition during electron flow process in the PSII system (**Figure 8**). Another photosynthesis parameter, *F*_v_/*F*_m_, may support our finding further (**Figure 9**). It is evident that environmental factors impacting the PSII, directly or indirectly, will also impact the *F*_v_/*F*_m_ values (Kumar et al., 2014). In the presence of light, abiotic and biotic stress often decrease *F*_v_/*F*_m_ in plants. Baker (Baker, 2008) suggested that *F*_v_/*F*_m_ measurement is as a simple and rapid way of monitoring stress in plants. Our results showed that *F*_v_/*F*_m_ decreased as prodigiosin treatment time increased, which provided evidence for PSII system dysfunction. This dysfunction transferred excitation energy to ROS as singlet oxygen. Chl *a* and carotenoid content decreased (**Figure 7**), reducing the inhibition capacity of ROS generation. Carotenoids are important antioxygenic micromolecule in photosynthesis organisms, which could quench the excited triplet state of chlorophyll, thus preventing excessive ROS generation (Larkum, 2016).

D1 protein, including in the reaction center of PSII, holds important redox components involved in the photosynthetic charge separation and subsequent reduction of plastoquinone (Mulo et al., 2012). Wu et al. (2014) had reported that the highly oxidative chemistry of water splitting could induce severely damage to the PSII system. The D1 protein is the main objective of this damage, but it could sacrify itself to block PSII inactivation. In other words, the D1 protein is constantly degraded and re-synthesized in the cycle called PSII repair cycle under normal photosynthetic conditions. Our results showed that algal cells treated with prodigiosin reduced *psb*A expression significantly, which suggests that the disturbance of D1 protein synthesis process inducing serious oxidative damage to PSII (**Figure 10**). RuBisCO, encoded by *rbc*S and *rbc*L genes, has important function in carbon fixation in plants and algae (Atkinson et al., 2015). The *rbc*S gene was first downregulated, and then upregulated at 24 h, indicating that cells try to fix more CO_2_ to resist external stress. The *rbc*S gene was also inhibited by prodigiosin as treatment time increased. These results indicate that the photochemical reaction in PSII and the carbon fixation system were completely inhibited by prodigiosin.

**FIGURE 11:**
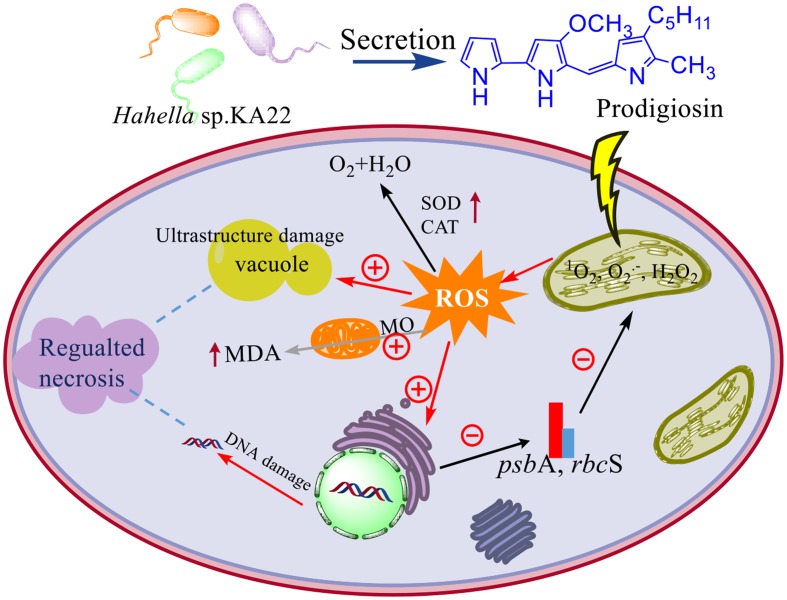
The model of algicidal mechanism of prodigiosin on *P. globosa*. ⊕ represents the promoted process; ⊖represents the inhibited process; the increased enzyme activity and the MDA contents were represented by the up arrow; MO means membrane oxidative damage.

Although prodigiosin was reported as an efficient algicidal compound years ago, this is the first repot exploring its algicidal mechanisms on *P. globosa*. Because of its high algicidal activity and natural degradation characteristics, prodigiosin has great potential as an algicide for the regulation of red tide occurrence. Therefore, based on the above analysis, we proposed the model of algicidal mechanism of prodigiosin on *P. globosa* (**Figure 11**). The model suggests that prodigiosin strongly inhibits photosynthesis, promoting excessive ROS generation, which may induce severe oxidative damage to cells and organelles. Our results indicate that algal cells undergo necrosis promoted by ROS when they are treated with prodigiosin. Further studies, including the analysis of algicidal effects on harmful algae present in a microcosm, are essential, prior to the application in the control of red tides.

## Author Contributions

HZ contributed for conception and design, drafting of the article, technical and logistic support, analysis and interpretation of the data. HW contributed for collection and assembly data and analysis the data. WZ, ZY, YP, and SZ contributed for statistical expertise and collection and assembly data. ZH and ZT contributed for critical revision of the article for important intellectual content. TZ contributed for obtaining of funding and final approval of the article. All authors had reviewed the manuscript.

## Conflict of Interest Statement

The authors declare that the research was conducted in the absence of any commercial or financial relationships that could be construed as a potential conflict of interest. The reviewer YB and handling Editor declared their shared affiliation, and the handling Editor states that the process nevertheless met the standards of a fair and objective review.
